# Glutathione restores the mitochondrial redox status and improves the function of the cardiovascular system in old rats

**DOI:** 10.3389/fphys.2022.1093388

**Published:** 2023-01-09

**Authors:** Nataliіa Strutynska, Yulia Goshovska, Lidiia Mys, Ruslan Strutynskyi, Alina Luchkova, Raisa Fedichkina, Iryna Okhai, Yuliia Korkach, Vadym Sagach

**Affiliations:** ^1^ Department of Blood Circulation, Bogomoletz Institute of Physiology, National Academy of Sciences of Ukraine, Kyiv, Ukraine; ^2^ Department of General and Molecular Pathophysiology, Bogomoletz Institute of Physiology, National Academy of Sciences of Ukraine, Kyiv, Ukraine

**Keywords:** aging, heart, vasorelaxation, ischemia, mitochondria, glutathione, hydrogen sulfide

## Abstract

**Introduction:** Aging is accompanied by cardiovascular disorders which is associated with an imbalance of pro- and antioxidant systems, the mitochondrial dysfunction, etc. Glutathione (GSH) plays a critical role in protecting cells from oxidative damage. The aim of the work was to study the effect of exogenous glutathione on the redox status of mitochondria, the content of H_2_S and the function of the cardiovascular system in old rats.

**Methods:** Experiments were performed on adult (6 months) and old (24 months) Wistar rats divided into three groups: adult, old and glutathionetreated old rats. Glutathione was injected intraperitoneally at a dose of 52 mg/kg. We investigated glutathione redox balance, H_2_S levels, oxidative stress, the opening of the mitochondrial permeability transition pore (mPTP), the resistance of isolated heart to ischemia/reperfusion in Langendorff model, endothelium-dependent vasorelaxation of isolated aortic rings, and cardiac levels of *3-MST*, *CSE*, and *UCP3* mRNA were determined using real-time PCR analysis.

**Results:** Our data shows that in old rats treated with glutathione, the balance of its oxidized and reduced form changes in the direction of a significant increase (by 53.6%) of the reduced form. Glutathione pretreatment significantly increased the H_2_S levels, mtNOS activity, and *UCP3* expression which considered as protective protein, and conversely, significantly decreased oxidative stress markers (the rate of O_2_•^−^ generation, the levels of H_2_O_2_, diene conjugates and malone dialdehyde, in 2.5, 2.3, 2, and 1.6 times, respectively) in heart mitochondria. This was associated with the inhibition mitochondrial permeability transition pore opening and increased resistance of the isolated heart to ischemia/reperfusion in these animals. At the same time, in glutathione-treated old rats, we also observed restoration of endothelium-dependent vasorelaxation responses to acetylcholine, which were almost completely abolished by the NO-synthase inhibitor L-NAME.

**Conclusion:** Thus, the pretreatment of old rats with glutathione restores the mitochondrial redox status and improves the function of the cardiovascular system.

## 1 Introduction

Glutathione is a low-molecular-weight thiol that is endogenously synthesized in cells from glutamic acid, cysteine, and glycine. There are two forms of glutathione—reduced (GSH) and oxidized (GSSG) in the cell. The ratio of these forms changes dynamically depending on metabolic processes and the level of oxidative stress. As a natural low-molecular antioxidant and electron donor, glutathione is involved in maintaining cellular homeostasis and plays a key role in protecting cells from oxidative stress, inhibiting apoptosis, and is also necessary for the detoxification of xenobiotics, drug metabolism, etc. Glutathione provides the efficient functioning of the number of proteins, including the electron transport chain, ATPase, ion channels, transporters, etc. (Cramer et al., 2017). Disturbance of the intracellular GSH/GSSG balance is associated with general signs of aging as well as with a wide range of pathological conditions of cardiovascular diseases, neurodegenerative disorders like Parkinson’s disease, etc. (Sekhar et al., 2011; Homma and Fujii, 2015). In addition, the increasing of oxidative and nitrosative stress in the heart during aging and the decreasing of hydrogen sulfide (H_2_S) concentration along with a decline of a constitutive synthesis of nitric oxide (NO) collectively lead to the dysfunction of mitochondrial mechanisms, in particular to the formation of a non-selective channels—the mitochondrial permeability transition pores (mPTP) in the high-conductance mode, which is the cause of many pathological conditions (Strutynska et al., 2016; Carraro et al., 2020; Matuz-Mares et al., 2022; Strutynska et al., 2022). Therefore, prevention of mPTP induction by inhibitors or antioxidants may be an effective way of cardioprotection.

It has been shown that administration of glutathione precursors has cardioprotective effects. Thus, N-acetyl-L-cysteine lowered blood pressure in hypertension (Tang et al., 1991), restored the content of GSH during myocardial infarction and heart failure in mice (Adamy et al., 2007). There is also a connection between glutathione and gaseous mediators such as hydrogen sulfide and nitric oxide, that are involved in the regulation of the functioning of the cardiovascular system. In particular, we observed cardioprotective effects of glutathione synthesis stimulation by D,L-propargylglycine and L-cysteine which was manifested in a significant recovery of the contractile function of the heart, increasing NO constitutive synthesis, and inhibition of ROS production in the ischemic myocardium of adult rats (Goshovska et al., 2021). The application of this combination on animals with diabetes also improved heart function and endothelium-dependent vasorelaxation (Dorofeyeva et al., 2021). We also showed that under aging conditions, the cofactor of H_2_S-synthesizing enzymes (pyridoxal-5-phosphate) restored eNOS coupling and endothelium-dependent vascular relaxation (Mys et al., 2020) as well as prevented mPTP induction (Mys et al., 2018) and improved heart function (Mys et al., 2022). On the other hand, in *CSE* knockout (*CSE−/−*) mice, a low-cysteine diet resulted in glutathione and H_2_S deficiency, elevated plasma homocysteine levels, and developmental delay and death at 12 weeks of age (Mani et al., 2011). All this indicates the important role of glutathione and H_2_S in the regulation of the cardiovascular system in aging and prevention of the pathological processes. Thus, literature data and our results indicate the importance of the reduced form of glutathione in heart and vascular cells for their antioxidant status and normal functioning of the cardiovascular system. The aim of the present work was to study the effect of exogenous glutathione in its reduced form on markers of mitochondrial oxidative stress, hydrogen sulfide content, and the function of the cardiovascular system in old rats.

## 2 Materials and methods

### 2.1 Animals

All procedures were conducted in accordance with the Directive 2010/63/EU of the European Parliament and of the Council on the protection of animals used for scientific purposes (22.09.2010). The experimental protocols were approved by the Biomedical Ethics Committee of the Bogomoletz Institute of Physiology National Academy of Sciences of Ukraine (No. 2/21, 16 June 2021). The experiments were performed on adult (6 months old, 220–250 g) and old (22–24 months old, 350–450 g) male Wistar rats. Animals were housed in a neutral temperature environment (22°C ± 2°C) on a natural day-to-night cycle with free access to water and on a standard diet. Rats were divided into three groups: adult, old and glutathione-treated old animals (old + GSH). Each group included 6–12 animals. Animals were anesthetized by intraperitoneal injection of urethane at a dose of 1.5 g/kg. For our study, we used sodium glutathione (Hepaval, Valartin Pharma LLC, Ukraine/Italy, powder for injections) which was diluted in the water for injection and injected intraperitoneally at a dose of 52 mg/kg 1 h before test preparations.

### 2.2 RNA isolation and real-time polymerase chain reaction (PCR) analysis

The total RNA was isolated from the heart apex tissue using Tri reagent (Sigma-Aldrich). The concentration and purity of total RNA were determined using a NanoDrop spectrophotometer ND1000 (NanoDrop Technologies Inc., United States). Reverse transcription was performed using 500 ng of RNA and the RevertAid First Strand cDNA Synthesis Kit (Thermo Fisher Scientific, United States). For *3-MST*, *CSE*, *UCP3* and beta-actin, RT-PCR amplification reaction was performed in a volume of 20 μl containing 0.9 μl Gene Expression Assay (Thermo Fisher Scientific), 10 μl TaqMan™ Fast Universal PCR Master Mix and 2 μl cDNA. Gene Expression Assay used for RT-PCR are presented in Table 1. PCR was performed for 50 cycles of 10 min at 95°C, 15 s at 95°C, and 60 s at 60°C using 7,500 Fast Real-Time PCR (Applied Biosystems, United States). The threshold cycle (Ct) was automatically calculated by instrument software. Then, ΔCt was calculated as the difference in Ct values of target genes and housekeeping gene, beta-actin. The relative gene expression was calculated as 2^−ΔΔCT^ (Livak and Schmittgen, 2001).

**TABLE 1:** Gene Expression Assay that were used to determine gene expression.

Gene	Product size	Accession number	Gene expression assay
*MPST* (*3-MST*)	68 bp	NM_138843.1	Rn00593744_m1
*CTH* (*CSE*)	66 bp	NM_017074.1	Rn00567128_m1
*UCP3*	80 bp	NM_013167.2	Rn00565874_m1
*ACTB*	91 bp	NM_031144.3	Rn00667869_m1

### 2.3 Determination of biochemical indicators

#### 2.3.1 Determination of glutathione levels in heart tissue

The measurements of oxidized (GSSG) and reduced glutathione (GSH) were performed in the heart homogenates with Ellman’s reagent (Rahman et al., 2006). Hearts were washed with cold (+4°C) KCl 0.9% solution, weighed and homogenized in isolation buffer based on 0.1 M potassium phosphate buffer (KPE) with the addition of 5 mM EDTA (EDTA sodium salt) at pH = 7.5, 0.1% Triton X-100% and 0.6% sulfosalicylic acid. The ratio of tissue to isolation buffer was 1:9. The homogenized tissue was centrifuged (Allegra X-22R, Beckman Coulter, United States) at +4°C, 8,000 g for 10 min. Next, the supernatant was leached into clean microtubes. An aliquot of supernatant for GSH measurement was immediately frozen (−20°C). The GSSG probe, 1 ml of supernatant was mixed with 30 μl of 97% 2-vinylpyridine in KPE (1:10), stirred, and 60 μl of 98% triethanolamine in KPE (1:6) was added after an hour, stirred again, and frozen. Measurements were performed using a Biosan HiPo MPP-96 microplate reader (Lithuania). 60 μl of 500 units glutathione reductase in KPE solution (1:150), 60 μl 0.8 mM cofactor β-NADPH and 60 μl 1.68 mM dithiobisnitrobenzoic acid were added to initiate the reaction. Optical density was measured immediately and for 2 min each 30 s at 405 nm. The concentrations of GSH and GSSG were calculated according to the linear regression equation obtained from the calibration curve of the standard GSH and GSSG solutions. The data were presented in grams per milligram of the examined tissue.

#### 2.3.2 Measurement of the H_2_S content

The content of H_2_S was determined in the suspension of heart mitochondria as described previously with modifications (Strutynska et al., 2022). For clarity, the term H_2_S will be used to represent all sulfides. In this case, the aliquot of the samples were immediately mixed with 0.5 ml of 1% zinc acetate to capture the maximum amount of sulfide in the biological material and incubated at 37.5°C for 10 min. Then, .5 ml of 20 mmol/L N,-N-dimethyl-p-phenylenediamine in 7.2 mol/L HCl and 0.5 ml of 30 mmol/L FeCl_3_ in 1.2 mol/L HCl solution were added. After 20 min incubation at 37.5°C, 0.5 ml of 10% trichloroacetic acid was added to the reaction mixture prior to the addition of 2.5 ml distilled water. The absorbance of the resulting solution at λ =670 nm was measured. The H_2_S concentration was calculated according to the calibration curve of the NaHS solution. The mitochondrial fraction was obtained from the rat heart by the method of differential centrifugation. The removed hearts were washed with a cooled 0.9% KCl solution. The heart tissue was minced and homogenized in the isolation medium in a ratio of 1:9, which contained (mmol/l): sucrose—250, EDTA—1, Tris-HCl—25, pH 7.4. To remove the nuclei and cell debris, the homogenate was first centrifuged at a low speed at 700 g for 8 min (2°С). The resulting supernatant was centrifuged at high-speed at 11,000 g for 15 min (2°C) to mitochondria precipitate, after which it was washed again. The mitochondrial precipitate was stored in a resuspension medium containing (mmol/l): sucrose—250 and Tris-HCl—25 (pH 7.2), and immediately used in the experiment or stored at low temperatures.

#### 2.3.3 Assessment of the oxidative stress markers and mtNOS activity in heart mitochondria

Biochemical indicators of oxidative stress such as the rate of formation of superoxide (O_2_•^−^) and hydrogen peroxide pools (H_2_O_2_) were measured in the suspension of heart mitochondria. Pools of diene conjugates (DC) and malondialdehyde (MDA) were measured as markers of lipid peroxidation. The methods used to assess oxidative stress markers and parameters of NO system were described in detail previously (Mys et al., 2020; Strutynska et al., 2022). Determination of mitochondrial NOS activity was performed by methods adapted for spectrophotometric measurement of one of the reaction products—L-citrulline. Enzyme activities were expressed as amount of L-citrulline generated for 1 min per 1 mg of protein in the sample. The concentration of L-citrulline was found from the calibration curves. The protein content in the samples of heart tissue homogenates was determined by the Lowry method.

### 2.4 Registration of mPTP opening in the heart of rats

Hearts were removed from decapitated rats, and washed with a cold 0.9% solution of KCl (4°C). Mitochondria were isolated by differential centrifugation and protein content was determined in organelle suspension by the method of Lowry. To record mPTP opening, the suspension of organelles was used for 2–4 h mPTP opening was investigated by spectrophotometric registration of the swelling of mitochondria isolated from the rat heart (Strutyns’ka et al., 2011). For this purpose, mitochondria were placed in the incubation medium of isotonic composition (mmol/L): KCl—120, Tris-HCl—25, KH_2_PO_4_—3, sodium succinate—5, pH 7.4, and a decrease in the optical density in mitochondria suspension was recorded at *λ* = 520 nm for 15 min of the mitochondria swelling. Protein concentration was 0.4 mg/ml. As a control, mitochondria suspension was used in the incubation medium in the absence of an inducer with the following registration of the optical density for 15 min. The amplitude (A) of spontaneous and Ca^2+^-induced swelling of the organelle suspension in a Ca^2+^-free medium and in the conditions of pore-formation activation with the action of PTP inducer of swelling of calcium ions was calculated as the difference in optical density values from 1 to 15 min mPTP-associated mitochondrial swelling was tested by the specific mPTP inhibitor cyclosporin A (CsA), which was added to the incubation medium in a concentration of 10^−5^ mol/L 5 min before the induction of pore formation. The results were processed using Origin software (“Microcall Inc.,” United States).

### 2.5 Langendorff isolated heart perfusion protocol

Langendorff isolated rat heart model was used to study myocardial resistance to ischemia/reperfusion (I/R). After the excision of the heart, it was mounted to the Langendorff apparatus. The perfusion of the coronary vessels was performed retrogradely under stable perfusion pressure of 75–80 mmHg with non-recirculating Krebs-Henseleit solution (in mmol/L): NaCl—118; KCl—4.7; MgSO_4_—1.2; NaHCO_3_—24; KH_2_PO_4_—1.2; glucose—10; CaCl_2_—2.5, pH = 7.4, 37°C, constantly aerated by gas mixture (95% O_2_ and 5% CO_2_). Each heart was allowed to stabilize for 20 min. After the stabilization period, the hearts were subjected to 20 min of total ischemia followed by 40 min reperfusion. Left ventricle developed pressure (LVDP), end-diastolic pressure (EDP), the first derivative of left ventricle pressure (dP/dt), and the heart rate were measured using a latex balloon placed into the left ventricle connected to the strain gauge (Elema, Sweden) and recorded using Global Lab software. The coronary flow rate was defined as the volume of solution passing through the heart for 1 min. The intensity of the contractile function (heart work) was calculated as the product of LVDP and heart rate. For the determination of arteriovenous difference, we measure the partial pressure of oxygen in the samples of the flowing in and out solution of the heart using a gas analyzer BMS3Mk2 (Denmark). Myocardial oxygen consumption was calculated according to Neely (Neely et al., 1967) considering coronary flow and heart dry weight, which averaged 0.212 g in adults and 0.237 g in old animals. The oxygen cost of myocardial work was expressed as the ratio of oxygen consumption to heart work.

### 2.6 Study of endothelium-dependent relaxation of isolated aortic rings

The experiments were performed on isolated aortic rings of rats perfused with Krebs solution saturated with a gas mixture (95% O_2_ and 5% CO_2_). All tests were done in isometric mode at the initial set voltage at which they generated maximum force in response to noradrenaline infusion (10 μmol/L). The temperature of the solution in the experimental chamber (37°C ± 0,5°C) was maintained using an automatic thermostat KISS 208B (Huber). Before measurement, the vascular strips fixed in the experimental chamber were kept for 60 min in Krebs solution of the following composition (in mmol/L): NaCl—120.4; KCl—5.9; NaHCO_3_—15.5; NaH_2_PO_4_—1.2; MgCl_2_—1.2; CaCl_2_—2.5; glucose—11.5. Relaxation of vascular strips was studied against the background of increased tone of aortic preparations obtained with the help of norepinephrine perfusion (10 μmol/L). We studied endothelium-dependent responses of vascular rings using acetylcholine which was introduced into the perfusing solution in doses from 0.1 to 10 μmol/L. The nature of endothelium-dependent vasodilatory responses was determined using an inhibitor of NO-synthases *N*_ω_-Nitro-L-arginine methyl ester hydrochloride (L-NAME) which was added to the perfusing solution at a dose of 100 μmol/L for 40 min and was administered 20 min before the introduction of acetylcholine.

### 2.7 Statistical analysis

The data were expressed as mean ± S.E.M. Shapiro-Wilk test was used to evaluate the normality of the distribution of data in each group. Comparison between groups was made using one-way analysis of variance (ANOVA) followed by *post hoc* Tukey HSD test or non-parametric Kruskal-Wallis test for multiple independent samples with *post hoc* test by the methods of Conover. *p* < 0.05 was assumed as statistically significant.

## 3 Results

### 3.1 Levels of total, reduced and oxidized glutathione in the heart tissue

**FIGURE 1:**
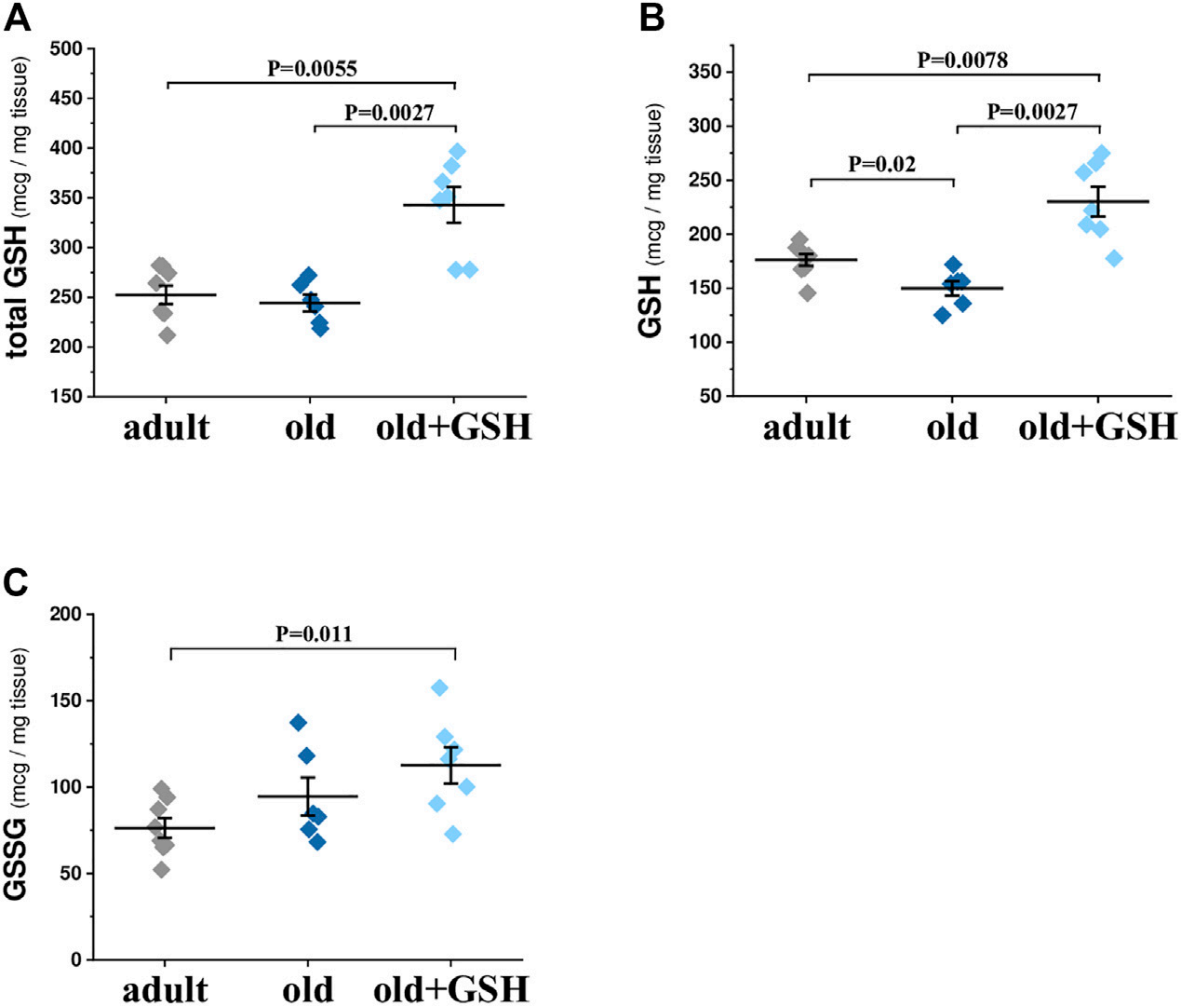
Levels of total **(A)**, reduced **(B)** and oxidized **(C)** glutathione in the heart tissue of adult, old and glutathione-treated old rats. GSH, glutathione. Data are represented as mean ± SEM; *n* = 7–8. Indicated *p* values were calculated by Kruskal-Wallis test.

The analysis of total glutathione content showed no difference between the heart of old and adult rats (Figure 1A). However, the balance of the oxidized and reduced forms of glutathione (GSH and GSSG) was changed in old rat hearts: the concentration of the reduced form was decreased by 14.9% (*р* = .02) and the levels of the oxidized form of glutathione were increased slightly in cardiac tissue of old rats (Figures 1B, C). Pretreatment with exogenous glutathione to old rats led to a significant increase in total glutathione content by 40% (*p* = 0.0027) compared to old untreated rats (Figure 1A). Also GSH level was significantly increased by 53.6% (*p* = 0.0027) compared to old control animals (Figure 1B). Notably, the content of GSSG was not significantly increased in old + GSH group comparing to old one (Figure 1C). Thus, increase in total glutathione content occurred due to increase in its reduced form that may at least partially participate in establishing of the redox status of cardiac tissue.

### 3.2 Oxidative stress markers, H_2_S level and mtNOS activity in heart mitochondria in glutathione-treated old rats

It was shown that in old rat heart mitochondria, the values of oxidative stress indicators such as generation rates of O_2_•^−^ and H_2_O_2_ as well as the content of the primary products of lipid peroxidation products (DC and MDA) were higher in heart mitochondria of old rats comparing to adult ones (Table 2) indicating aggravation of free radical processes with age. In particular, the rate of O_2_•^−^ generation was higher in 3.7 times in the heart mitochondria of old animals (*p* < 0.05), however, glutathione pretreatment lowered the generation rate of O_2_•^−^ in 2.5 times (*p* < 0.05). Administration of glutathione to old rats also reduced H_2_O_2_ generation rate in 2.3 times (*p* < 0.05) which was 2-fold higher in old rats than in adults (Table 2). The contents of DC and MDA in old heart mitochondria were higher in 4.3 and 2.8 times (*p* < 0.05) compared to adults. The administration of glutathione decreased these indicators twice for DC and in 1.6 times for MDA (*p* < 0.05 for both) (Table 2). Therefore, a decrease of oxidative stress markers in heart mitochondria under the conditions of administration of glutathione to old animals shows the antioxidative effects of this compound and the important role of sufficient levels of GSH in the maintenance of redox status of old rat heart mitochondria.

**TABLE 2:** Oxidative stress markers and mitochondrial NO synthase (mtNOS) activity in heart mitochondria of adult, old and GSH-treated old rats.

	Adult (*n* = 9)	Old (*n* = 9)	Old + GSH (*n* = 9)
O_2_•^−^, (nmol/min/mg protein)	4.62 ± 0.22	16.87 ± 4.87*	6.70 ± 0.38*^,#^
H_2_O_2_, (pmol/mg protein)	12.36 ± 3.11	25.59 ± 3.24*	11.06 ± 0.10^#^
MDA, (nmol/mg protein)	2.11 ± 0.15	5.93 ± 0.36*	3.71 ± 0.20^#^
DC, (ng/mg protein)	3.73 ± 0.24	16.01 ± 2.25*	7.70 ± 0.92^#^
mtNOS activity, (pmol/min/mg protein)	4.5 ± 0.24	2.28 ± 0.39*	8.62 ± 2.05^#^

Note: GSH, glutathione; O_2_•^−^, Superoxide; H_2_O_2_, hydroperoxide; MDA, malondialdehyde; DC, diene conjugates.

*р < 0.05 versus adult.

^#^ *p* < 0.05 versus old as calculated by ANOVA, test.

The content of hydrogen sulfide was decreased 3.6 times in old rat heart mitochondria compared to adults (*p* < 0.05). At the same time, glutathione-treated old rats showed the restored level of H_2_S to the values of the adult group (Figure 3D). Also, mtNOS activity decreased almost twice in old animals compared to adults (*p* < .05) but it was almost doubled in old + GSH group (*p* < 0.05) (Table 2). Thus, exogenous glutathione improved the production of gaseous transmitters (NO and H_2_S) which are important regulators of not only the redox status of the cells but also calcium transients and mitochondrial membrane permeability.

### 3.3 mPTP identification and sensitivity of mPTP to calcium in the heart of glutathione-treated old rats

**FIGURE 2:**
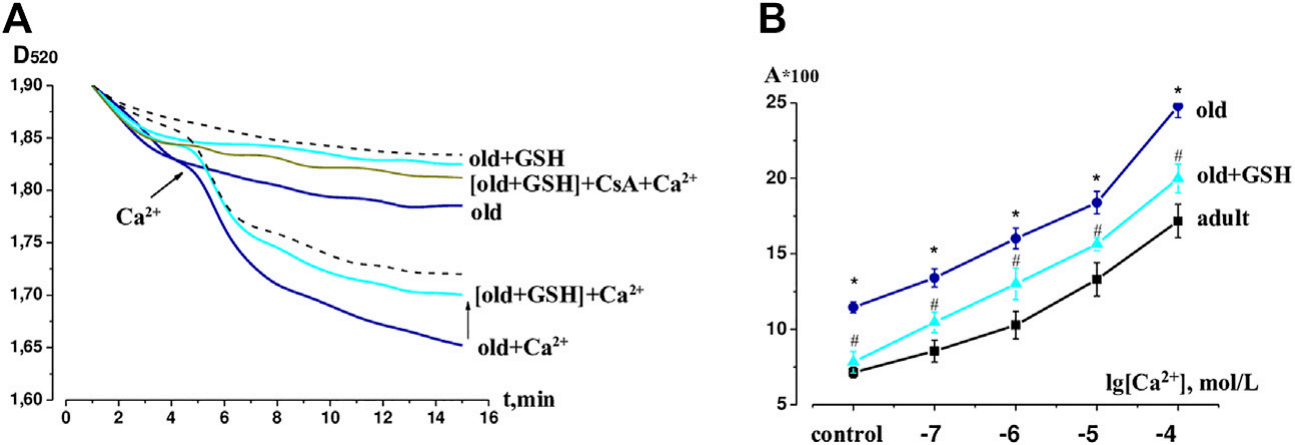
The effects of GSH pretreatment on the mPTP in heart of old rats. **(A)** Native curves of Ca^2+^-induced mitochondrial swelling. **(B)** Changes in the sensitivity of mPTP to calcium ions (10^−7^–10^−4^ mol L^−1^) in the heart of adult, old and GHS pretreated old rats: the ordinate axis shows the difference between the observed amplitude of swelling of mitochondrial swelling on the 15th min and the optical density of suspension on the 1st min. The dashed line shows the swelling of the adult mitochondria in Ca^2+^-free and Ca^2+^-contained mediums. GSH, glutathione. Data are represented as mean ± SEM; *n* = 7–9. *р < 0.05 versus adult; ^#^ *р* < 0.05 versus old as calculated by ANOVA test.

Figure 2 shows typical kinetic curves of spontaneous mitochondrial swelling in Ca^2+^ free medium and under conditions of calcium loading as well as changes in sensitivity of mitochondria to a wide range of Ca^2+^ concentration under our study conditions. The amplitude of the spontaneous and Ca^2+^-dependent swelling in old rat heart mitochondria significantly exceeded such indicators in adult animals by 60.4% and 44.4% (*р* < 0.05 for both) respectively. The effect of glutathione pretreatment of old rats was manifested in a significant lowering of the mitochondrial swelling amplitude compared to untreated old rats both in a Ca^2+^-free environment and under the effect of Ca^2+^ by 84.3% (*р* < 0.05) and 63.1% (*р* < 0.05) respectively and approached the values of adult animals (Figure 2A). Preincubation of heart mitochondria of GSH-treated old animals with inhibitor CsA (10^−5^ mol/L) almost completely prevented Ca^2+^-induced mitochondrial swelling, which indicates the association of this process with mPTP opening (Figure 2A).

Under the conditions of Ca^2+^ influence in the concentration range of 10^−7^–10^−4^ mol/L, a dose-dependent swelling of mitochondria isolated from rat heart tissue was observed (Figure 2B). The sensitivity of mPTP to Ca^2+^ was evaluated by calculating the amplitude of control and Ca^2+^-dependent swelling of heart mitochondria in the studied groups of animals. The upward shift of the concentration curve indicated an increase in mPTP sensitivity to the inducer in old rats relative to adult animals, however, in old + GSH group, we observed a significant decrease in mPTP sensitivity to Ca^2+^. Thus, our data proves the mitoprotective properties of exogenous glutathione pretreatment.

### 3.4 Effect of glutathione administration on *3-MST*, *CSE* and *UCP3* expression in heart

**FIGURE 3:**
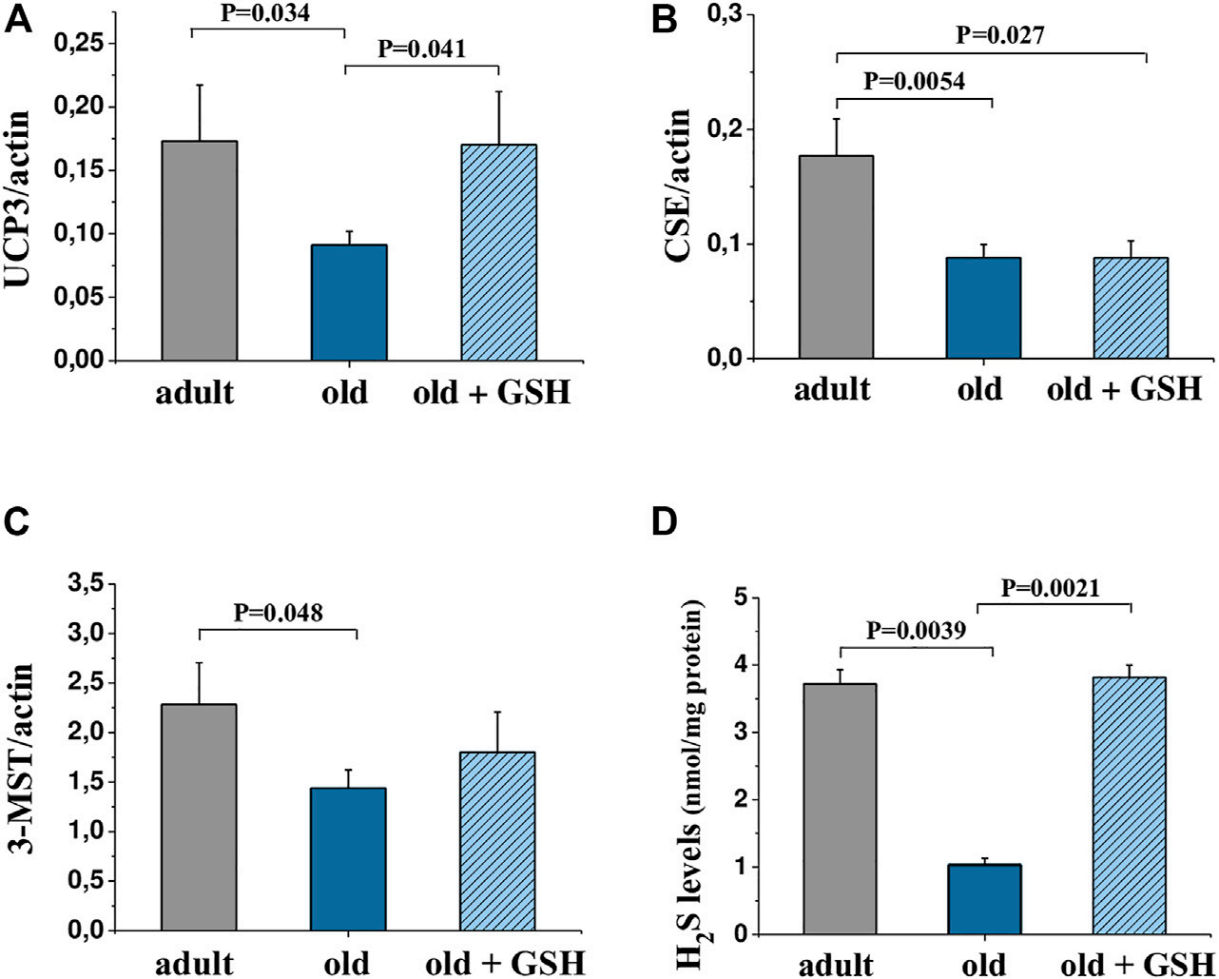
mRNA levels of genes encoding *UCP3* **(A)**, *CSE* **(B)** and *3-MST* **(C)** in the heart tissue and H_2_S levels in heart mitochondria **(D)** of adult, old and glutathione-treated old rats. GSH, glutathione. Data are represented as mean ± SEM; *n* = 7–9. Indicated *p* values were calculated by Kruskal-Wallis rank sum test.

The determining of the levels of mRNA expression of *UCP3* in the heart of adult and old rats did show a significant decrease in expression of *UCP3* in old rat hearts compared to the adult, *p* = .034 (Figure 3A). Pretreatment with glutathione restored the expression levels of *UCP3* in old rats to the level of adult animals (Figure 3A) increasing the *UCP3* expression levels in 1.87 times compared to old (*p* = 0.041). The levels of expression of H_2_S-synthesizing enzymes *CSE* and *3-MST* were also significantly lower in old rats compared to adults (Figures 3B, C), however, glutathione pretreatment did not change the expression of genes of these enzymes (Figures 3B, C).

### 3.5 Cardiodynamics and oxygen metabolism of isolated heart in glutathione-treated old rats

Basic cardiodynamics of the isolated heart is shown in Table 3. First of all, it should be noticed the significant differences between adult and old animals in the values of the LVDP and the myocardial contraction force (dP/dt max) that were increased by 36.2% and 29.8% respectively (*p* < 0.001 for both). At the same time, heart work increased only by 14.2% (n/s) that conditioned by the decrease in heart rate by 20.6% (*p* < 0.01).

**TABLE 3:** Effect of GSH pretreatment at cardiodynamics of isolated heart of old rats.

	Adult (*n* = 12)	Old (*n* = 12)	Old + GSH (*n* = 6)
Left ventricle developed pressure, (mmHg)	110.1 ± 4,8	150.0 ± 6,5***	169.6 ± 7.2
dP/dt max, (mmHg/s)	2129 ± 124	2765 ± 140***	3265 ± 188
dP/dt min, (mmHg/s)	−1963 ± 85	−2112 ± 91	−1955 ± 295
Coronary flow, (ml/min)	10.4 ± .5	15.8 ± 1.4**	13.3 ± 1.3
Heart rate, (bpm)	261 ± 9.7	207 ± 12**	186 ± 8
Heart work, (rel. un.)	26820 ± 1360	30640 ± 1899	31389 ± 1466
Oxygen consumption, (×10^−3^ mmol O_2_ per min per g of tissue)	4.34 ± 0.36	7.20 ± 0.46**	4.97 ± 0.27^##^
Oxygen cost of myocardial work, (×10^−7^ mmol О_2_ per min per g of tissue per rel. un.)	1.54 ± 0.14	2.43 ± 0.23**	1.55 ± 0.07^#^

Note: GSH, glutathione. **p < 0.01, ***p < 0.001 versus adult.

^#^ *p* < 0.05.

^##^ *p* < 0.01 versus old as calculated by Kruskal-Wallis test.

The process of myocardial relaxation (diastole) depends on the rate of pumping of calcium ions from the cytoplasm by calcium pumps (SERCA) into the sarcoplasmic reticulum and the efficiency of ATP synthesis by mitochondria. The rate of relaxation of the myocardium (dP/dt min) was the same in both adults and old animals which with increased heart size (by 11.7%, based on dry weight) and increased coronary flow (by 51.9%) indicates a violation of dilatation functions. At the same time, the oxygen consumption by the myocardium and the oxygen cost of myocardial work increased significantly (by 65.9% and 57%, respectively, *p* < 0.01 for both). The obtained results are typical for old animals (Goshovska et al., 2009) and indicate calcium overload of cardiomyocytes, energy deficit, and intensification of oxidative metabolism and support the data about the excessive formation of ROS in aged rat heart mitochondria (Table 2).

Intraperitoneal administration of glutathione to old rats did not significantly affect either the contractile force of the isolated heart or coronary flow but significantly reduced oxygen consumption and the oxygen cost of the heart to the level in adult rats, *p* < 0.01 for both (Table 3). Thus, exogenous glutathione may improve the non-effective effect oxygen utilization by aged myocardium probably effecting mitochondrial function and metabolism.

### 3.6 The effect of glutathione on the reperfusion recovery of the function of the ischemic heart of old rats

**FIGURE 4:**
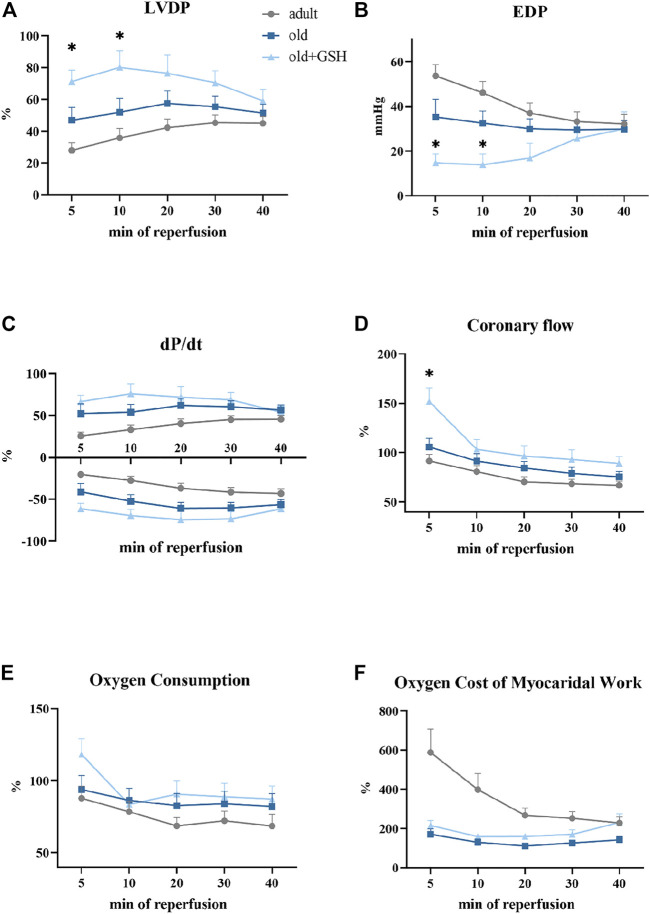
Effect of glutathione pretreatment on cardiodynamics restoration of the isolated rat heart after 20 min total ischemia: the left ventricle developed pressure **(A)**, the end-diastolic pressure **(B)**, the first derivative of the left ventricle pressure, dP/dt **(C)**, the coronary flow **(D)**, the oxygen consumption **(E)**, the oxygen cost of myocardial work **(F)**. Data are represented as mean ± SEM; *n* = 12 in the adult, *n* = 12 in old, *n* = 6 in old + GSH groups. GSH, glutathione. *p < 0.05 versus old as calculated by Kruskal-Wallis test.

We compared the restoration of heart function upon reperfusion after 20 min of total ischemia. For this purpose, all data were converted to percentages (except for EDP) where the preischemic values were taken as 100%. We observed significant depression of heart contractile function in adults and old animals as low percentage of LVDP and dP/dt recovery after ischemia (Figure 4). Also, the dilatation of myocardium was damaged by I/R and manifested as a sharp rize of EDP values at the 5th min of reperfusion (Figure 4B).

Administration of glutathione to old rats slightly improved recovery of the contractile function of the isolated heart especially in the early period of reperfusion. In old + GSH group, at the 5th min of reperfusion, LVDP was 71% from the baseline level compared to 46.8% in the old group (*p* < 0.05). At the 10th min of reperfusion, this indicator was 80.2% compared to 51.8% in the old rats (*p* < 0.05) (Figure 4A). At the 5th min of reperfusion, EDP was 14.6 mmHg compared to 35.2 mmHg in old, *p* < 0.05 (Figure 4B). Importantly, the coronary flow was in 1.5 times higher at 5th min of reperfusion in old + GSH group than in old ones, *p* < 0.05 (Figure 4D). Although there were no significant differences in the rate of contraction and relaxation (dP/dt) of the myocardium (Figure 4C), the dynamics of these parameters were slightly better in the old + GSH group than in untreated old. At the same time, in old treated animals, the dynamic of oxygen cost of myocardial work did not differ from the untreated group (Figure 4E) that might be due to compensatory mechanisms in myocardium developed with aging.

Despite some positive effects of GSH pretreatment on resistance of old myocardium to I/R, the improvement of heart function recovery was not observed in the late period of reperfusion that may indicate exhausted intrinsic antioxidant reserves of the myocardium, so further studies are needed for the investigation of long-term increase of myocardial resistance to I/R.

### 3.7 Effect of glutathione administration on endothelium-dependent relaxation of isolated vascular rings

**FIGURE 5:**
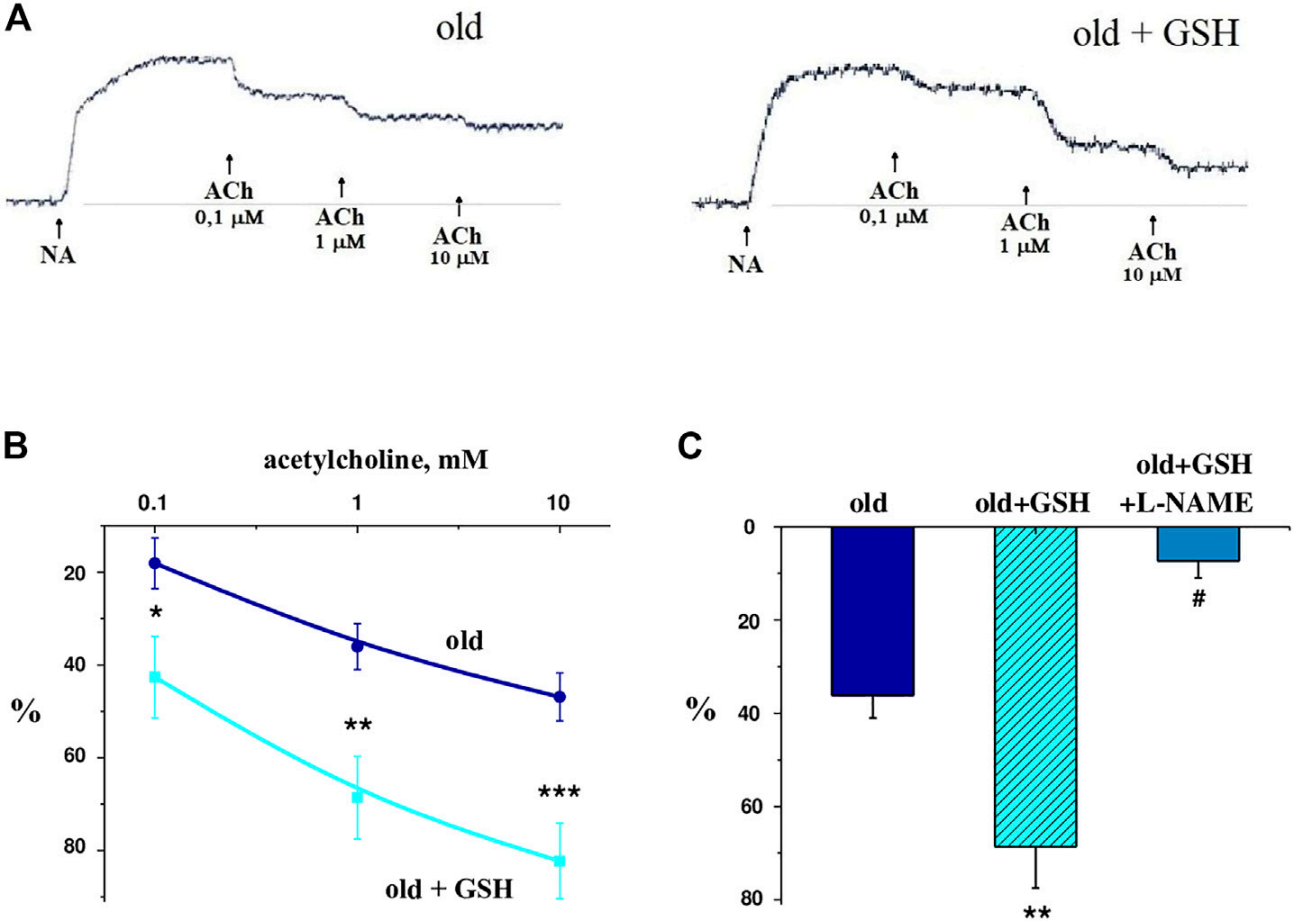
Vascular preparations relaxation in response to different doses of acetylcholine against the background of norepinephrine-induced vasoconstriction of GSH-treated and untreated old rats: **(A)** native curves; **(B)** dose-dependent curves; **(C)** relaxation of isolated aorta rings in response to acetylcholine (1 μmol/L) in old rats, old GSH-treated and old GSH-treated rats background of L-NAME (100 μmol/L). Data are represented as mean ± SEM; *n* = 7–8. *р = 0.047 versus old; **р = 0.017 versus old; ***р = 0.0077 versus old; ^#^ *р* = 0.0026 versus old + GSH as calculated by ANOVA test.

Figure 5A shows typical records of relaxation curves of vascular preparations due to different doses of acetylcholine application against the background of norepinephrine-induced vasoconstriction of glutathione-treated and untreated old rats. It was shown that in old glutathione-treated rats, the reactions of relaxations were significantly higher in amplitude compared to untreated old rats (Figure 5B), in particular in 2.36 (*p* = 0.044), 1.9 (*p* = 0.017) and 1.75 times (*p* = 0.008) with an application of 0.1, 1 and 10 μmol/L acetylcholine respectively. The introduction of the NO-synthase inhibitor L-NAME (1 μmol/L) into the perfusing solution 20 min before the acetylcholine reduced the vasodilator responses of aortic rings to acetylcholine in 9.41 times (*p* = 0.003) (Figure 5C). These data indicate the NO-dependent nature of the vasodilatory response in old + GSH group. Thus, exogenous glutathione may improve vasorelaxation *via* activation of NO signaling pathway which is known to be impaired with aging.

## 4 Discussion

Glutathione is present in cells either in the form of GSH or in oxidized GSSG where two cysteine residues are linked by a disulfide bond. In physiological conditions, GSSG takes 1% of general glutathione in most cell types. It is recirculated by glutathione reductase with NADPH which is supplied by pentose phosphate pathway. The redox balance of glutathione affects the redox status of cells, subcellular structures and cellular homeostasis in general. It is known that depletion of GSH is caused by inhibition of its synthesis, lack of the amino acid L-cysteine, or knockdown of glutathione peroxidase 4. The deficit of GSH may result in a type of cell death called “ferroptosis.” The application of an iron chelator almost completely reverses ferroptosis which can be explained by the prevention of intralysosomal iron accumulation and leads to the suppression of induced oxidative stress permeabilization of lysosomal membranes (Homma and Fujii, 2015). Increased bioavailability of reduced glutathione provides antioxidant protection to mitochondria through the reduction of hydrogen peroxide and lipid hydroperoxides by enzymes of the glutathione peroxidase family (GPx1, GPx4). Another mechanism of GSH protection is reverse post-translational modifications of proteins by S-glutathionylation/deglutathionylation), which is common for the Krebs cycle and the electron transport chain proteins catalyzed by glutathione-S-transferase and glutaredoxins (Marí et al., 2020). Temporary S-glutathionylation of complex I inhibit its activity and ultimately limits the formation of ROS by the electron transport chain (Mailloux, 2020). At the same time, maintenance of the membrane potential of mitochondria is provided by complex II and requires glutathionylation of succinate dehydrogenase (Chen et al., 2007). Thus, a sufficient level of endogenous GSH is an important factor of protein functionality.

In this work, we showed that intraperitoneal administration of exogenous glutathione restored mitochondrial redox status and improved the function of the cardiovascular system of old rats. Interestingly, during aging, the total content of glutathione in the heart tissue was practically unchanged, but the balance of oxidized and reduced forms of glutathione changed in the direction of increasing GSSG (Figure 1). It is important that exogenous GSH not only significantly increased the content of total glutathione (by 40%) but also increased the endogenous content of its reduced form in cardiac tissue, thus contributing to maintenance of the redox potential of mitochondria and cardiac cells in general. As a result, we have found a decrease in O_2_•^−^ and H_2_O_2_ production as well as lipid peroxidation markers (DC and MDA) in heart mitochondria of old glutathione-treated rats (Table 2). Also, the oxygen cost of myocardial work of isolated old rat hearts pretreated with GSH was significantly decreased (Table 3) which means more effective oxygen utilization by myocardium and usage of oxygen for ATP production, not for ROS. Therefore, exogenous GSH provided a significant suppression of oxidative stress which indicates the antioxidant effects of this compound.

It is known that there is a tight relationship between H_2_S and GSH. H_2_S has been shown to increase intracellular levels of GSH that protected the cells from oxidative stress (Kimura et al., 2010). In our study, we showed the opposite connection: exogenous GSH increased H_2_S level (Figure 3D) that also had an antioxidative effect manifested in the reduction of oxidative stress markers in the heart mitochondria of old rats (Table 2). In our opinion, the increase in hydrogen sulfide production in glutathione-treated old animals was due to an increase in the expression of *3-MST* (by 25%), or probably the activity of this mitochondrial enzyme due to an excess of the substrate L-cysteine, which is common for the synthesis of this gaseous mediator and glutathione. The biologically active molecule H_2_S has strong reducing abilities, quenching ROS and reactive nitrogen species, providing protein sulfhydration, modulating cellular levels of glutathione and thioredoxin-1, increasing expression of antioxidant enzymes, etc. (Testai et al., 2016; Spassov et al., 2017; Corsello et al., 2018; Powell et al., 2018; Strutynska et al., 2022). Therefore, H_2_S may act as an antioxidant and together with glutathione may contribute to the reduction of oxidative stress.

Additionally, the decrease in plasma H_2_S in patients is now considered as marker of cardiovascular disorders like hypertension and coronary heart disease (Jiang et al., 2005; Feng et al., 2017). Li et al. (2022) showed that branched-chain amino acids were accumulated in the myocardium of *3-MST* KO mice which was accompanied by a decrease in mitochondrial respiration and ATP synthesis. This led to an exacerbation of cardiac and vascular dysfunction. Mitochondrial H_2_S produced by *3-MST* may play a regulatory role in branched-chain amino acid catabolism and contribute to critical cardiovascular protection in heart failure (Li et al., 2022). Although we observed only a tendency to increase in *3-MST* gene expression in old + GSH group, the increase in mitochondrial H_2_S content may occur due to upregulation of 3-MPST or *CSE* activity or/and *de novo* synthesis of H_2_S from L-cysteine derived from exogenous GSH.

Pretreatment with exogenous GSH affected not only H_2_S but also NO system. In our study, mtNOS activity in heart mitochondria was upregulated by pretreatment with exogenous glutathione which is definitely a positive effect. During aging, mtNOS switches to uncoupled mode, that involved loss of the ability to convert L-arginine into L-citrulline with the release of NO, instead, it catalyzes a transfer of electrons from NADPH to molecular oxygen with the formation of a superoxide anion radical (Roe and Ren, 2012; Strutynska et al., 2016). Reduced NO levels contribute to the development of mitochondrial dysfunction since mitochondrial NO as well as mitochondrial H_2_S directly inhibit Ca^2+^-induced mPTP opening (Strutyns’ka et al., 2011; Akopova et al., 2005). As the effect of exogenous glutathione, restoration of H_2_S level and mtNOS activity (and theoretically NO level) reduced the opening of the mPTP in the heart of old rats both in a Ca^2+^-free and Ca^2+^-containing medium (Figure 2A). Also pretreatment with glutathione significantly decreased sensitivity of mPTP to calcium (Figure 2B) which could indicate increased or restored Ca^2+^ capacity of mitochondria of old rat heart. Thus, glutathione-mediated preservation of mitochondrial function, in our opinion, became possible due to the reduction of ROS and restoration in mitochondria of the synthesis of gas mediators—NO and H_2_S which are important natural regulators of mPTP.

As a result of the reduction in oxidative stress and increase in H_2_S levels and mtNOS activity, we observed improved endothelium-dependent relaxation which dramatically deteriorated with aging (Brandes et al., 2005; Mys et al., 2020). Intolerance of vessels to vasorelaxants like acetylcholine led to permanently increased tone of smooth muscle cells and age-associated progression of arterial hypertension. That is why antioxidative medications are mandatory components of anti-hypertensive therapy (Ahmad et al., 2017). In our study, restoration of redox status with exogenous glutathione was accompanied with improved vessel reactions to acetylcholine that seems to work in a NO-dependent manner since inhibitor of NOS (L-NAME) abolished the effect of GSH pretreatment.

Such positive changes in the redox status of the myocardium increase its resistance to ischemic effects. In general, ischemia-reperfusion of an isolated heart is accompanied by significant ultrastructural disorders of the myocardium, an increase in coronary perfusion pressure, rhythm disturbances during reperfusion, a decrease in the contractile function of the heart, etc. (Strutyns’kyĭ et al., 2008). In our experiments, in glutathione-treated animals, the indicators of recovery of ischemic heart at the early stage of reperfusion were significantly higher than those of control old animals. Thus, they had better reperfusion recovery of myocardial contractile activity (restoration of pressure in the left ventricle), increased coronary flow and efficiency of oxygen consumption (Figure 4).

Another interesting mechanism of the antioxidant effect of GSH may lay *via* the activation of cardiac uncoupling proteins (UCPs) (Figure 3A) that mediate H^+^-leak from intramembrane space of mitochondria into the matrix regulating ATP production *via* so-called “mild” uncoupling (Hirschenson et al., 2022). At certain conditions like cardiac I/R, this leakage reduces O_2_•^−^ production by the electron transport chain, thus, UCPs are considered protective proteins against oxidative stress (Cadenas, 2018; Nicholls, 2021). It is known that the expression and activity of UCPs are very sensitive to changes in the redox status of the cell and the concentration of oxygen in the cytoplasm (Zhou et al., 2000; Echtay et al., 2002). In our study, there was a restoration of *UCP3* mRNA level that is in agreement with reduced oxidative stress markers in GSH-pretreated old rat hearts. Together with other changes that we have found, this might be a reason for the improved resistance of the old rat heart to I/R that we observed. If there is upregulation of *UCP3* in cardiac tissue due to GSH pretreatment, further studies are needed to evaluate mitochondrial membrane potential and respiration of mitochondria; but we can speculate that *UCP3* may be one of the mechanisms involved in oxidative stress reduction by exogenous GSH.

Thus, here, we showed that exogenous glutathione has the potential to restore the redox status of cardiac mitochondria due to the reduction of oxidative stress, improvement of mtNOS activity and H_2_S levels which leads to the inhibition of mPTP and improvement of the functions of the cardiovascular system in old rats. Application of exogenous glutathione might be a useful approach for the correction of not only cardiovascular disturbances but also other systems’ function alteration associated with aging and oxidative stress.

## 5 Conclusion

Therefore, the mitochondrial dysfunction observed in aging is accompanied by the intracellular GSH/GSSG imbalance, a decrease of the H_2_S content in organelles and the expression levels of *UCP3*, as well as the uncoupling of mtNOS with an increase in ROS production, disruption of Ca^2+^ homeostasis, etc., which leads to the induction of mPTP with high conductance and causing cardiac dysfunction. Pretreatment of old animals with the reduced form of glutathione improved the functioning of the cardiovascular system, namely, restored endothelium-depended relaxation of aortic rings, decreased oxygen cost of myocardial work and increased myocardial resistance of myocardium to I/R. The data we obtained indicate that when exogenous glutathione is administered to old rats, such mechanisms of protection and stabilization can develop as 1) an increase in the content of the reduced form of glutathione in heart tissues and, thus, improvement in the redox status of heart tissues and mitochondria; 2) suppression of ROS production and lipid peroxidation processes in mitochondria; 3) restoration of mtNOS activity and H_2_S level that contributes to the regulation of CsA-sensitive mPTP opening and mitochondrial Ca^2+^ homeostasis; 4) activation of mitochondrial *UCP3* as a component of antioxidant protection. These results reflect the important role of mitochondria redox status in the functioning of the cardiovascular system, as well as the possibility of its restoration in aging by the introduction of exogenous glutathione.

## Data Availability

The original contributions presented in the study are included in the article/Supplementary Material, further inquiries can be directed to the corresponding author.
